# Deletion of the *Bcnrps*1 Gene Increases the Pathogenicity of *Botrytis cinerea* and Reduces Its Tolerance to the Exogenous Toxic Substances Spermidine and Pyrimethanil

**DOI:** 10.3390/jof7090721

**Published:** 2021-09-03

**Authors:** Ana Fernández-Morales, María Carbú, Victoria E. González-Rodríguez, Sokratis Papaspyrou, Carlos Garrido, Jesús M. Cantoral

**Affiliations:** 1 Laboratory of Microbiology, Department of Biomedicine, Biotechnology and Public Health, Faculty of Marine and Environmental Sciences, University of Cadiz, 11510 Puerto Real, Spain; maría.carbu@uca.es (M.C.); victoriaeugenia.gonzalez@uca.es (V.E.G.-R.); carlos.garrido@uca.es (C.G.); 2 Department of Biology, Faculty of Marine and Environmental Sciences, University of Cadiz, 11510 Puerto Real, Spain; sokratis.papaspyrou@uca.es

**Keywords:** non-ribosomal peptide synthetase, environmental signals, polyamines, fungicide, RT-qPCR, siderophore, oxidative stress

## Abstract

During the infection of grapevine (*Vitis vinifera*) by the fungus *Botrytis cinerea*, the concentration of polyamines, which are toxic substances for the phytopathogen, increases in the grape. Nine NRPS genes have been identified in the genome of *B. cinerea*, yet the function of five of them remains unknown. For this reason, we have studied the expression of the 9 NRPS genes by RT-qPCR in a medium supplemented with sublethal concentrations of three polyamines (1,3-diaminopropane (1,3-DAP), spermidine (SPD), and spermine (SPM)). Our results show that the presence of polyamines in the culture medium triggered the overexpression of the *Bcnrps*1 gene in the pathogen. Deleting *Bcnrps*1 did not affect mycelial growth or adaptation to osmotic stress, and we show that its expression is not essential for the cycle of infection of the *B. cinerea*. However, mutating the *Bcnrps*1 gene resulted in overexpression of the *Bcnrps*6 gene, which encodes for the excretion of siderophores of the coprogen family. Moreover, gene deletion has reduced the tolerance of *B. cinerea* B05.10 to toxic substances such as the polyamine SPD and the fungicide pyrimethanil, and its virulence has increased. Our findings provide new insights into the function of the *Bcnrps*1 gene and its involvement in the tolerance of *B. cinerea* against exogenous toxic compounds.

## 1. Introduction

*Botrytis cinerea* (teleomorph *Botryotinia fuckeliana*) is a phytopathogenic fungus belonging to the phylum Ascomycota and is the causal agent of gray mold disease. It can infect over 200 ornamental and agriculturally important plant species, resulting in considerable economic losses [1,2,3]. *B. cinerea* infects and kills host cells by producing and releasing many secondary metabolites such as virulence factors, toxins, and reactive oxygen species [4].

Sequencing and annotation of the complete genome of *B. cinerea* [2,5] revealed more than 40 clusters of genes implicated in the synthesis of polyketides, terpenes, and non-ribosomal peptides. However, to date, only eight families of secondary metabolites have been identified in vitro [2,6].

The described non-ribosomal peptides are a diverse family of biologically active natural compounds, including toxins, siderophores, pigments, antibiotics (e.g., daptomycin), cytostatics, immunosuppressants (e.g., cyclosporin), and anticancer agents (e.g., bleomycin) [7,8,9]. In fungi, non-ribosomal peptides can also contribute to tolerance to oxidative stress [10]. The synthesis of non-ribosomal peptides is carried out by the non-ribosomal peptide synthetase (NRPS). Nine NRPS genes have been identified in the latest update of *B. cinerea’s* genome [2,5]. Three of these annotated genes (*Bcnrps*2, *Bcnrps*3, *Bcnrps*7) are involved in the biosynthesis of intracellular siderophores [11,12]. In contrast, another one (*Bcnrps*6) may code for an enzyme responsible for the biosynthesis of a siderophore belonging to the coprogens family [13,14,15]. The role of the five remaining genes is yet to be clarified.

Our understanding of the functions that remaining genes play is hindered by the fact that many secondary clusters are not activated under in vitro conditions since they require environmental cues produced during pathogen-host interaction [16]. During plant-pathogen infection, reprogramming of metabolism occurs in the host plant in response to the interaction with the pathogen. When the plant defense system is activated, it triggers the production of different hormones, such as salicylate, jasmonate, and ethylene, and antimicrobial secondary metabolites such as phytoalexins [16,17]. On the other hand, several studies report that the biosynthetic pathways of polyamines (PAs) are relevant for responding to biotic and abiotic stress [18,19,20,21,22]. The most common PAs present in plant tissue are spermine (SPM), spermidine (SPD), and putrescine (PUT), followed by cadaverine (CAD) and 1,3-diaminopropane (1,3-DAP) [23].

When *B. cinerea* infects *Vitis vinifera*, the plant’s transcriptome is reprogrammed, and thus, different pathways are up- or down-regulated compared to healthy plants [24]. The PAs pathways are up-regulated in plants to fight against the infection [25,26]. Furthermore, previous studies with filamentous fungi describe the activation of NRPS biosynthetic pathways by the addition of 1,3-diaminopropane and spermidine to the culture medium [27].

We asked whether the presence of PAs in the culture media could modify the expression of *Bcnrps* genes to elucidate their role in the fungi’s life cycle. For this purpose, we studied the expression of NRPS genes in *B. cinerea* by RT-qPCR in the presence and absence of three polyamines. Furthermore, we deleted the NRPS gene with the highest expression under the study conditions, and we characterized the mutant in terms of its growth capacity, metabolic activity, tolerance to oxidative stress and exogenous toxics substances, siderophores production, and pathogenicity.

## 2. Materials and Methods

### 2.1. Strain and Culture Conditions

The *B. cinerea* B05.10 strain was used in all experiments. This strain has been isolated from a *Vitis vinifera* in California (USA) and was a kind gift of Dr. Muriel Viaud of the UMR BIOGER, INRA (Grignon, France). For routine culturing, the strain was grown on potato dextrose agar (PDA) medium at 24 °C. The conidia necessary for fermentation were obtained by growing the fungus on agar-tomato medium (500 mL of blended tomato, 0.1% malt extract, and 2% agar). Fermentations used for expression study were carried out in malt extract medium (ME: 0.2% malt extract, pH 6.5–7) supplemented with three different PAs: 1,3-DAP (Sigma-Aldrich, St. Louis, MO, USA), SPM (Sigma-Aldrich), and SPD (Sigma-Aldrich). The strains were cultured on Petri dishes containing solid synthetic complete medium (CM) [28] to determine and quantify siderophore assay and maintenance of knockout strains. Synthetic medium (SM) [29] was used in the sensitivity to fungicide test.

### 2.2. Determination of Sublethal Concentrations of Polyamines in B. cinerea Cultures

Erlenmeyer flasks containing 50 mL of ME medium were inoculated with 10^5^ conidia/mL of *B. cinerea* B05.10 and supplemented with increasing amounts (0, 0.5, 1, 1.5, 2, 2.5, 5, 10, 15, 20, 25 y 30 mM) of three polyamines (PAs): 1,3-DAP, SPM, and SPD, one per flask. Flasks were incubated at 25 °C, under orbital shaking at 120 rpm, and 15/9 h light/dark cycles for 72 h. ME medium inoculated with the conidia and without the addition of PAs was used as a positive control. Assays were carried out in triplicate. A sublethal concentration was considered to be the maximum concentration of polyamine that allowed spore germination and mycelial development of *B. cinerea*.

### 2.3. Fermentation of B. cinerea in the Presence or Absence of PAs

Erlenmeyer flasks containing 50 mL of ME medium without or with the optimal sublethal PAs concentration were inoculated with 10^5^ conidia/mL of *B. cinerea* B05.10 strain. All cultures were incubated at 25 °C, under orbital shaking at 120 rpm, and 15/9 h light/dark cycles for 22 days. The mycelium was harvested 4, 8, 12, 19, and 22 days post inoculation (dpi), preserved in RNAlater^®^ (Invitrogen, Waltham, MA, USA), and frozen until performing RNA extractions. All fermentations were carried out in triplicate using independent conidia samples for inoculation.

### 2.4. Primers Design for Non-Ribosomal Peptide Synthase Encoding Gene Amplification

The sequences of the nine genes encoding NRPS enzymes in the genome of *B. cinerea* B05.10 strain as well as of the β-tubulin (*BctubA*) and actin (*BcactA*) genes were retrieved from the Ensembl Fungi database. The *BctubA* and *BcactA* genes are commonly used as housekeeping genes in RT-qPCR expression studies in *B. cinerea* and other fungi [30] and were selected to normalize expression values (Table S1). The SeqBuilder software package was used to design specific primers for qPCR. The criteria used for primer design were as follows: 50% G + C content, a length of 20 nucleotides, and amplicon sizes between 100 and 150 bp (Table S1). Genomic DNA was extracted from the *B. cinerea* B05.10 strain using the protocol described by Garrido et al. [31]. Conventional PCR was performed with each pair of primers, and the specificity of the primers sets was confirmed visually by obtaining a single amplification product on a 1% (*w*/*v*) agarose gel electrophoresis.

### 2.5. RNA Extraction and cDNA Synthesis

Each frozen mycelium was first homogenized in 2 mL tubes containing 450 mg glass-beads 425–600 μm (Sigma-Aldrich), two 1/4″ CeramicSpheres (Q-Biogene, Carlsbad, CA, USA), and 1 mL of Trizol reagent (Invitrogen) using a Fast-Prep Instrument (2 cycles: 6 m/s for 30 s) (Fast-Prep^®^-24, MP Biomedicals, Santa Ana, CA, USA). Then RNA was extracted as described by Froehlich et al. [32]. RNA was purified using RQ1 RNase-Free DNase (Promega, Madison, WI, USA), and total RNA purity and concentration were determined using a NanoDrop^TM^ 2000c spectrophotometer (Thermo Scientific, Waltham, MA, USA). For quantitative RT-qPCR expression analyses, 1 µg of RNA of each sample was reverse-transcribed into cDNA using the PrimeScriptTM RT Reagent Kit (Perfect Real Time) (Takara, San Jose, CA, USA) according to the manufacturer’s protocol. Control reactions were performed without reverse transcriptase to verify the absence of gDNA contamination.

### 2.6. Real-Time PCR Assays

All real-time PCR amplifications were carried out using the CFX96 Touch™ Real-Time PCR Detection System (BioRad, Hercules, CA, USA) and Maxima SYBRGreen/ROX qPCR Master Mix (2×) kit (Thermo Scientific) following the manufacturer’s instructions. Primer concentrations were optimized, creating a 4 × 4 reaction matrix, using a range of 100–400 nM for each primer against different concentrations of the partner primer. Annealing temperatures were also optimized, testing a range from 54 to 62 °C. For each experiment, efficiency curves were drawn for each pair of primers; a negative control was used, and each reaction was repeated in three technical replicates.

Amplification conditions consisted of an initial step for enzyme activation at 50 °C for 2 min, a denaturation step at 95 °C for 10 min, and 44 cycles at 95 °C for 15 s and 1 min at 60 °C. Melting curve analysis was set up from 65 to 95 °C, with an increment of 0.5 °C for 5 s and plate read. Relative transcriptomic expressions of the *Bcnrps* genes were analyzed statistically following Gil-Salas et al. [33]. This method calculates the mean normalized expression (MNE) using the average Ct value from the three replicates for the target gene, the average for the normalizer gene and their corresponding intercepts and slopes from calculated standard curves. The variance and standard error were also calculated. All tests were carried out using the three experimental replicates per treatment.

### 2.7. Bioinformatic Analyses of Bcnrps1 Gene

Orthologous genes were identified in the database Ensembl Fungi developed by the EMBL-EBI [34] (European Molecular Biology Laboratory and the European Bioinformatics Institute). BlastP was used to search the protein sequence of NRPS1 against all fungal genomes available in the National Center for Biotechnology Information from the U.S. National Library of Medicine (NCBI) [35] using the Blast web tool. Protein domains in the *Bcnrps*1 sequence were identified using the Conserved Domain Database (CDD) of NCBI [36] and the Ensembl Fungi database [34]. In addition, the Non-ribosomal Peptide Synthase Substrate Predictor database [37] was used to predict the specific amino acid for the adenylation domain, which determines the scaffold of the metabolite.

### 2.8. Generation of Bcnrps1 Mutant

Inactivation of the *Bcnrps*1 gene was carried out by means of protoplast generation and homologous recombination. The 5′ and 3′ flanking and noncoding regions of the *Bcnrps*1 gene were amplified from genomic DNA using the primer pairs *Bcnrps*1-5F/*Bcnrps*1-5R and *Bcnrps*1-3F/*Bcnrps*1-3R (Table S2) respectively and assembled with the hygromycin (*hph*) resistance cassette by yeast recombinant cloning [38]. The hygromycin resistance cassette was amplified with the primer pairs *hphF-trpC*-P/*hphR-trpC*-T (Table S2) using the pCSN44 vector as a template.

Protoplast generation and transformation were performed as described previously by Schumacher [39]. The transformed protoplasts were regenerated and selected on SH agar medium containing 70 mg/mL hygromycin B (Invitrogen, Leek, The Netherlands) as the selection agent. Resistant colonies were transferred to Petri dishes containing Gamborg’s B5 medium (Duchefa Biochemie BV, Haarlem, The Netherlands) [39] supplemented with 2% glucose, 0.1% yeast extract, and the selection agent at the same concentration used before. Homologous integration of the NRPS1 replacement fragment events in hygromycin-resistant transformants was confirmed by diagnostic PCR using the primers *TrpC*-P2 and *TrpC*-T binding in the HygR cassette, and the primers *Bcnrps*1-Hi 5′ and *Bcnrps*1-Hi 3′ binding upstream and downstream of the *Bcnrps*1-flanking regions, respectively (Table S2). Single spore isolates were screened for the absence of wild-type alleles by using primers *Bcnrps*1-WT-F and *Bcnrps*1-WT-R (Table S2) matching the substituted coding region. The length of the *Bcnrps*1 gene prevented the construction of a complementation cassette. The absence of ectopic integrations was checked by Southern blot analyses. Fungal genomic DNA was digested with restriction enzyme *BamH*I (Fermentas, Baden-Wurttemberg, Germany), separated on 1% (*w*/*v*) agarose gels, and transferred to Amersham Hybond-NX membrane (GE Healthcare Limited, Buckinghamshire, UK). Blot hybridization with Amersham Gene Images AlkPhos Direct Labeling and Detection System (GE Healthcare Limited, UK) were performed according to the manufacturer′s instructions. The blot was hybridized with the 3′ flank of the replacement fragment.

### 2.9. Characterization of the Bcnrps1 Mutant

#### 2.9.1. Study of the PAs Effect on Metabolic Activity and on the Growth of Strains

A resazurin (RZ) microtiter assay was used to determine the effect of the sublethal concentration of each PAs on the metabolic activity of NRPS mutant (Δ*Bcnrps*1) against the wild type (WT). In each well, 100 μL of the PAs solution in ME medium, 80 µL of a suspension of 10^5^ conidia/mL, and 20 µL of 400 µM RZ were added. Four wells per plate were used for each treatment. As a negative control, wells contained culture medium and RZ without the conidia suspension for each PA. In addition, a positive control to determine the capacity of conidia to reduce RZ without polyamines was also added. The plate was incubated for 12 h, at 25 °C, under continuous shaking, and absorbance measurements were performed at 570 and 600 nm.

Metabolic activity of the fungal conidia was measured as the percentage reduction in the RZ as a consequence of the respiration rate of the fungus. In the presence of metabolically active cells, RZ passes from its oxidized state (blue) to the reduced state (pink). The color variation can be measured spectrophotometrically, allowing to determine the metabolic activity of fungal conidia. RZ reduction increases as the metabolic activity is higher and was calculated using the following equation described by Vega et al. [40]:

$$RZ\;\left(\%\;reduction\right)\;=\;\frac{\left(\left(\varepsilon ox\right)\lambda 2A\lambda 1-\;\left(\varepsilon ox\right)\lambda 1A\lambda 2\right)\;}{\left(\varepsilon red\right)\lambda 1A^{\prime }\lambda 2-\;\left(\varepsilon red\right)\lambda 2A^{\prime }\lambda 1)}\;\times 100$$

where *εox* = molar extinction coefficient of RZ oxidized form (blue; 80.586 and 117.216 for 570 and 600 nm, respectively); *εred* = molar extinction coefficient of RZ reduced form (pink; 155.677 and 14.652 for 570 and 600 nm, respectively); *A* = absorbance of test wells; *A*′ = absorbance of negative control wells; *λ*1 = 570 nm; and *λ*2 = 600 nm.

To determine the effect of PAs on the growth of the mutant strain compared to WT strain, a mycelium disk was placed on a plate with ME medium supplemented with the sublethal concentration of 1,3-DAP, SPM, and SPD. As a control, the strains were grown under the same conditions without supplementing the culture medium with PA. The plates were incubated at 25 °C under natural light for 4 days, and the growth areas were measured using the JMicroVision 1.2.7 software. The effect on growth was expressed as the mean of the growth area with respect to control without PAs. All tests were carried out in triplicate.

#### 2.9.2. Effect of SPD on the Production and Excretion of Siderophores

CAS blue agar, used in siderophores determination and quantification, was prepared according to Pérez-Miranda et al. [41].

To determine the production capacity of siderophores, culture plates were prepared with a bottom layer of CAS blue agar [42] and a layer of CM medium supplemented with SPD added on top. The plates were inoculated in the center with 10 µL of a 10^4^ conidia/mL suspension of WT or mutant strains in distilled water and incubated at 25 °C under continuous darkness. The plate area that turned from blue to yellow because of iron (III) removal by the siderophores excreted by the fungus was measured with the JMicroVision 1.2.7 software [43]. All tests were carried out in triplicate.

The concentration of excreted siderophores was quantified against a calibration curve (R and R^2^ > 0.99) using the standard commercial deferoxamine mesylate B (DFMB) siderophore, following the methodology optimized by the research group, which is in the process of publication.

#### 2.9.3. Sensitivity Test to Oxidative Stress and Exogenous Toxic Substances

Mycelial plugs (5 mm diameter) from a 4-day-old colony were transferred to plates with ME medium supplemented with 5 or 10 mM hydrogen peroxide [44] and incubated for 4 days at 25 °C in continuous darkness.

The sensitivity test to an exogenous toxic substance was performed following the same methodology and conditions as the oxidative stress test described above, except that SM was used as the culture medium [29]. The substance chosen was the fungicide pyrimethanil PESTANAL^TM^ (Sigma-Aldrich), whose maximum sublethal concentration for the WT strain has been established at 1 µg/mL. The effect of pyrimethanil in the WT and knockout strains were recorded at 6 and 3 dpi.

The growth areas were measured using JMicroVision 1.2.7 software. All tests were carried out in triplicate.

#### 2.9.4. Pathogenicity Assays

The study of the infectivity of the mutant strain compared to the WT strain was performed following the protocol described by Zhang et al. [45]. Wounded pieces of apple (*Malus domestica*) and white grape berries (*Vitis vinifera*) were inoculated with 15 µL of a 10^5^ conidia/mL suspension of WT or mutant strains in potato dextrose broth (Condalab). Infected berries and apples were incubated in plastic boxes at 20 °C under natural illumination conditions. The infection process was monitored and documented photographically. The area of the lesions produced was measured at 4 dpi on apple and 9 dpi on white grape berry using the JMicroVision 1.2.7 software.

### 2.10. Statistical Analysis

The data were analyzed using *t*-test or ANOVA and Tukey post hoc using the R software version 3.6.2 [46], and the least significant difference at the 5% level was used to compare the means.

## 3. Results

### 3.1. Determination of Sublethal Concentration of PAs in B. cinerea Cultures

**Figure 1:**
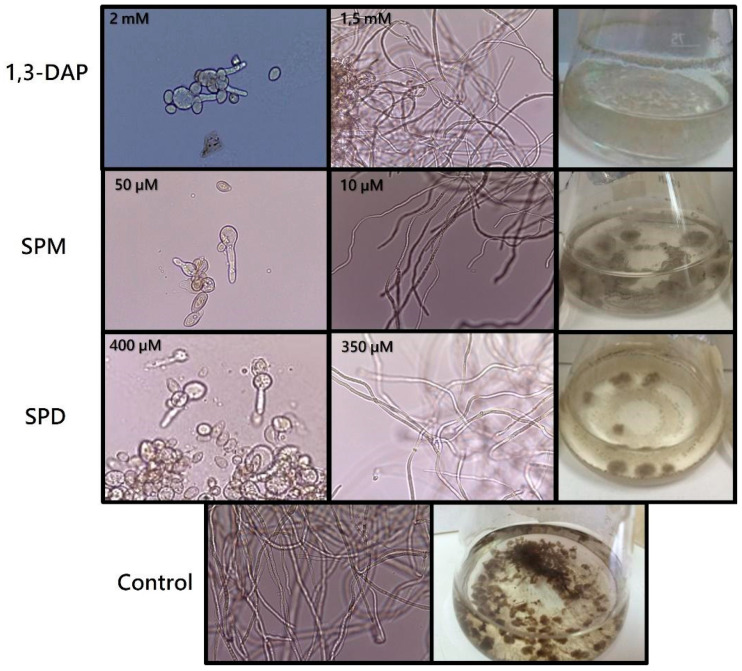
Determination of the sublethal concentration of polyamines in *B. cinerea* B05.10. Inhibition of spore germination at the highest concentration tested (1,3-DAP 2 mM; SPM 50 µM; SPD 400 µM). Hyphal growth at the sublethal concentrations of each polyamine (1,3-DAP 1.5 mM; SPM 10 µM; SPD 350 µM) and the control (0.2% malt extract medium). Growth of the fungus in liquid culture medium, showing the demelanization effect of each of the PAs on the mycelium with respect to the control. Conidia (400×) and mycelium (100×) were viewed under an optical microscope.

Fermentations with increasing concentrations of each agent (0, 0.5, 1, 1.5, 2, 2.5, 5, 10, 15, 20, 25, and 30 mM) showed that, in the presence of 1,3-DAP at 2 mM (Figure 1), spores of *B. cinerea* B05.10 did not germinate. In comparison, at 1.5 mM, both germination and mycelial development occurred. However, the strain was unable to grow at the concentrations of SPM and SPD tested initially. For this reason, new lower concentrations were analyzed (0, 10, 50, 100, 150, 200, 250, 300, 350, 400, 450 µM) for these polyamines. In the presence of SPM, the fungus developed a mycelium up to 10 µM, whereas in the presence of SPD, its development was possible up to 350 µM (Figure 1). Differences were also observed in the melanization of the mycelium of the fungus in the presence or absence (control) of PAs (Figure 1), with the highest degree of demelanization occurring in the presence of 1.5 mM 1,3-DAP.

### 3.2. Expression Profiles of Non-Ribosomal Peptide Synthetase Encoding Genes during Fermentation

#### 3.2.1. Real-Time PCR Optimization

Conventional PCR performed with each pair of primers and using genomic DNA or cDNA as a template confirmed the correct specificity and selectivity of each pair of primers. For real-time PCR amplification, optimal primer concentrations and annealing temperature were established at 300 nM and 60 °C, respectively. Standard curves were constructed by serial dilution of cDNA samples, and linear regression data demonstrated suitable efficiencies (>94%) for all the genes used (Table S3). Both *actin* and *β-tubulin* genes showed stable expression levels during the fermentation assays.

#### 3.2.2. Expression Profiles of *Bcnrps* Genes in the Absence and Presence of Polyamines

**Figure 2:**
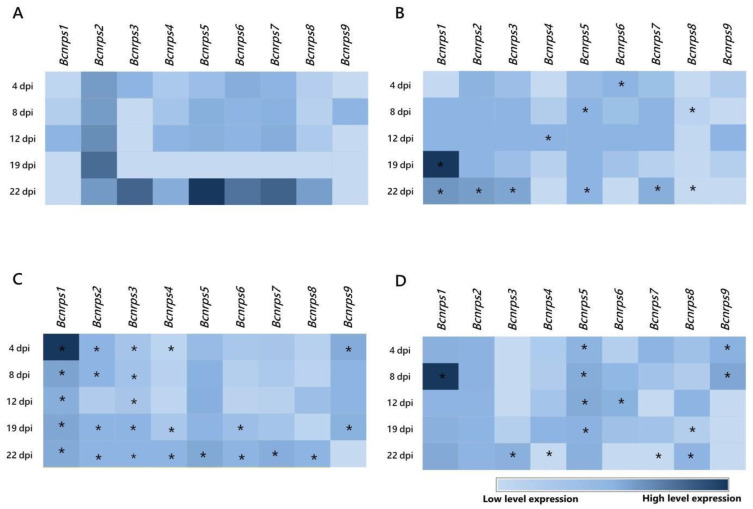
Heatmap of the expression profiles of the 9 *Bcnrps* genes during fermentation of *B. cinerea* B05.10 strain in the absence and presence of polyamines. The mean normalized expression (MNE) shown here is an average of the two MNE estimated using *actin* and *β-tubulin* as reference genes. (**A**) Malt extract medium (control), (**B**) malt extract supplemented with 10 µM SPM, (**C**) malt extract supplemented with 350 µM SPD, and (**D**) malt extract supplemented with 1.5 mM 1,3-DAP. Asterisks indicate significant differences of the 9 genes expressions between each supplemented medium and ME medium without PAs on different days of the study, according to two-way ANOVA, post hoc Tukey′s test for the factors media type and dpi (* *p*-value < 0.05).

During fermentation in ME medium without PAs (Figure 2A), the *Bcnrps*5 gene showed the highest level of expression, 22 dpi, followed by genes *Bcnrps*3, *Bcnrps*7, and *Bcnrps*6. Under these conditions, *Bcnrps*2 displayed the most stable levels of expression during the 22 days of the assay. The most remarkable difference between the *Bcnrps*2 and the other genes occurred 19 dpi when this gene showed its peak expression levels, whereas the rest showed low expression levels. In contrast, the *Bcnrps*1 and *Bcnrps*9 genes showed the lowest level of expression throughout the fermentation.

When the ME medium was supplemented with 10 µM of SPM (Figure 2B), the *Bcnrps*1 gene reached the highest level of expression 19 dpi, although with a variable expression profile during the 22 days of fermentation. The presence of SPM modified the expression profile of *Bcnrps*2 and *Bcnrps*5 genes with respect to the control, showing moderate expression levels throughout fermentation. In addition, at 22 dpi, the expression of *Bcnrps*3, *Bcnrps*6, and *Bcnrps*7 genes was affected, showing low to moderate expression levels. In the rest of the genes, the addition of PA did not produce an increase in gene expression.

The addition of SPD in the medium caused an increase in the expression of the *Bcnrps*1 gene, with a maximum recorded 4 dpi (Figure 2C). The rest of the genes showed similar expression profiles with low to moderate levels during the fermentation. Consistent with previous results, the addition of the polyamine SPM resulted, at 22 dpi, in decreased expression of the genes *Bcnrps*3, *Bcnrps*5, *Bcnrps*6, and *Bcnrps*7 compared to the expression in the control (Figure 2A).

In the fermentation with 1,3-DAP (Figure 2D), *Bcnrps*1 showed the highest expression level at 8 dpi. The expression of *Bcnrps*2 and *Bcnrps*5 were constant over time at moderate levels of expression. During the first days of fermentation (4 and 8 dpi), the expression of *Bcnrps*9 increased, while on the rest of the days, there was no difference in expression compared to the control (Figure 2A). The rest of the genes presented a variable expression profile during fermentation.

**Figure 3:**
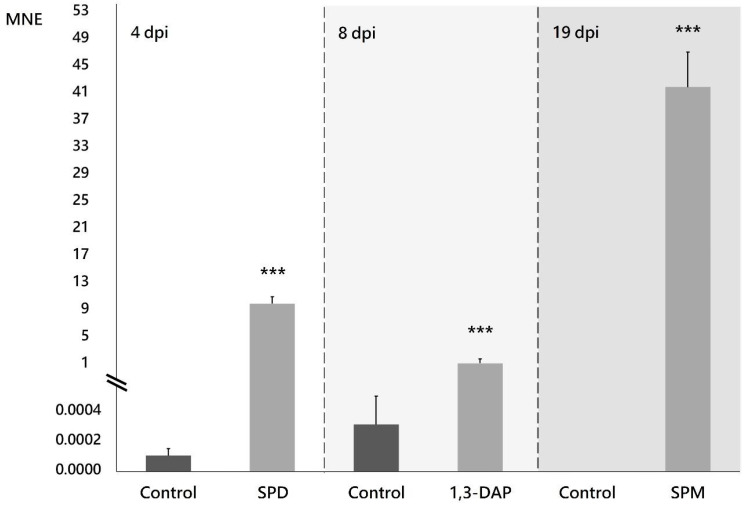
Expression of the *Bcnrps*1 gene (MNE) of *B. cinerea* B05.10 on the days when the highest expression levels were recorded in PAs-supplemented medium compared to control. Malt extract fermentation without PAs (control) is represented with gray bars, and fermentation supplemented with PAs with light gray bars. dpi: days post inoculation. Error bars represent standard deviations of means (*n* = 3). Asterisks indicate significant differences, according to *t*-test (*p*-value < 0.001, ***).

Supplementing the culture medium with the PAs selected in this study modified the expression profile of the NRPS family genes in *B. cinerea* B05.10 (Figures S1–S3), with the most notable effect on the expression of the *Bcnrps*1 gene. There was a statistically significant (*p* < 0.001) increase in the expression 4, 8, and 19 dpi in the presence of SPD, 1,3-DAP, and SPM, respectively (Figure 3). The most remarkable increase in *Bcnrps*1 expression was recorded in the presence of SPM 19 dpi (MNE SPM: 41.75) (Figure 3). While the presence of SPD (MNE SPD: 9.78) and DAP (MNE DAP: 1.42) (Figure 3) also caused overexpression of the gene compared to control conditions (ME), the effects were less noticeable.

### 3.3. Bioinformatic Analyses of Bcnrps1

Based on the results from the expression study, a bioinformatics analysis of the *Bcnrps*1 gene was performed to elucidate its putative function in *B. cinerea*. The analysis of the *Bcnrps*1 gene sequence (4885 bp) showed two orthologous genes from *Sclerotinia sclerotium* (target % ID: 84.94%; query % ID: 89.64%) and *Sclerotinia borealis* F-4128 (target % ID: 85.78%; query % ID: 86.37%) [33,34] (Table S4). However, the BLASTP analysis of the NRPS1 protein (1592 aa) indicated significant alignments (percent identity >80%; *e*-value = 0) with other NRPS in *B. cinerea* T4 and DW1 strains and with other species of *Botrytis* (Table S5), whose functions are still unknown.

Interestingly, the domains identified in the NRPS1 sequenced revealed that this protein is a mono-modular NRPS. Using the Conserved Domains Search Service of NCBI [37], we identified the conserved domains of NRPS1 (Table S6). The NRPS1 protein showed all the canonical NRPS domains, including a peptide synthase (cl36106) (interval 123–1148 aa), condensation domain (cd19537) (interval 1157–1549 aa), amino acid adenylation domain (cd17653) (interval 580–1048 aa), and phosphopantetheine-binding domain (pfam00550) (interval 32–95 aa). The analysis of the adenylation domain using the database Non-ribosomal Peptide Synthase Substrate Predictor tools [38] let us identify the putative substrate as phenylalanine. In addition, the NRPS1 protein contains a chloramphenicol acetyltransferase-like domain (IPR023213) (Table S6). This domain belongs to a superfamily that catalyzes the acetyl-CoA-dependent acetylation of chloramphenicol, thus inactivating the antibiotic [47].

### 3.4. Generation and Characterization of Bcnrps1 Mutant

To study the function of the *Bcnrps*1 gene in *B. cinerea*, a deletion strain was generated by replacing this gene with a hygromycin resistance cassette (P*trpC::hph*). After monoconidial strains, isolation, the integration of the replacement constructs was verified by PCR. Diagnostic PCR (Figure S4) revealed the homologous integration of the construct and homokaryotic strains in two independent transformants (T1.2 and T1.4). The absence of the ectopic integration was first tested by restriction enzyme analysis, producing larger hybridizing fragments in the replacement mutants (5.6 kb) than in the wild type (5.2 kb) and confirmed by Southern blot.

#### 3.4.1. Study of PAs Effect on Metabolic Activity and on the Growth of Strains

**Figure 4:**
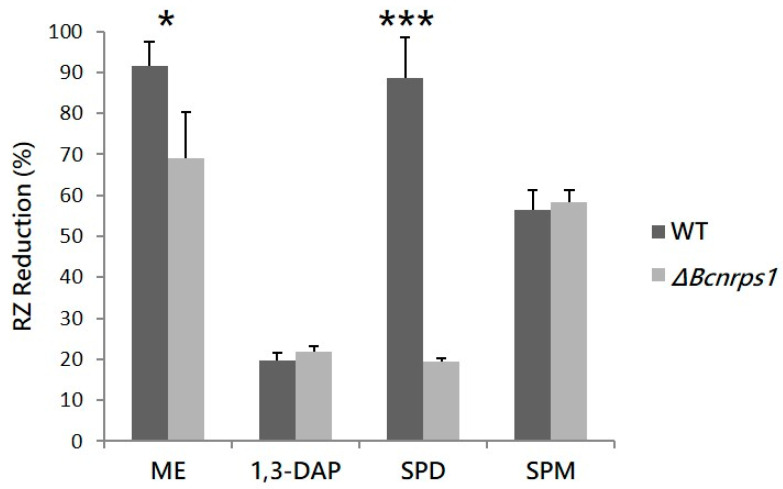
RZ reduction (%) due to the metabolic activity of the WT strain (gray) and the Δ*Bcnrps*1 mutant (light gray) 12 h post inoculation. ME: RZ reduction (%) in the positive control (0.2% ME medium); 1,3-DAP, SPD and SPM: RZ reduction (%) in ME medium amended with 1.5 mM, 10 µM and 350 µM of each PA, respectively. Error bars represent standard deviations of means (*n* = 3). Asterisks indicate significant differences between strains (WT and Δ*Bcnrps*1) when grown in medium supplemented with or without PAs, according to two-way ANOVA, post hoc Tukey′s HSD test for factors strain and media type (*p*-value < 0.05, *; *p*-value < 0.001, ***).

The effect of *Bcnrps*1 gene deletion on the metabolic activity of *B. cinerea* B05.10 conidia was quantified in the presence and absence of each polyamine, using the resazurin (RZ) microtiter test 12 h post inoculation (hpi). The WT strain produced a 94.8% ± 6% reduction in RZ in ME medium (Figure 4). In the presence of PAs, the metabolic activity of the conidia (93.5% ± 10%) was not affected by the addition of SPD to the medium. However, metabolic activity decreased 4.8- and 1.7-fold in the presence of SPM (56.5% ± 4.8% RZ reduction) and 1,3-DAP (19.6% ± 1.9% RZ reduction), respectively (Figure 4). The mutant strain displayed a significantly reduced metabolic activity in ME culture medium without PAs, showing an RZ reduction of 69.1% ± 11.2% (*p*-value < 0.05), whereas in the presence of SPD, this was as much as four times lower than for the WT strain (19.5% ± 0.6% RZ reduction; *p*-value < 0.001). In contrast, RZ reduction was not significantly different between the WT and Δ*Bcnrps*1 strains in culture media supplemented with SPM and 1,3-DAP (Figure 4).

**Figure 5:**
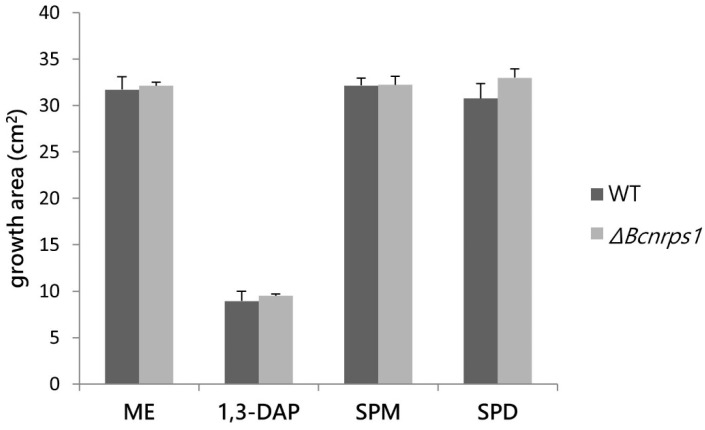
Influence of PAs on the growth area of the *B. cinerea* B05.10 strain (WT. gray) and Δ*Bcnrps*1 (light gray) 4 days post inoculation. ME: growth area in 0.2% ME medium without amended; 1,3-DAP, SPM, and SPD: growth area in ME medium amended with 1.5 mM, 10 µM, and 350 µM of each PA, respectively. Error bars represent standard deviations of means (*n* = 3). No significant difference was found between strains or media type (*p* > 0.05).

The effect of PAs on the growth of the WT *vs*. Δ*Bcnrps*1 strains was determined by studying the growth area in ME medium (control) with respect to ME medium supplemented with PAs at the optimal sublethal concentration established in this study (1.5 mM 1,3-DAP, 350 µM SPD, or 10 µM SPM). None of the three PAs tested had a cytotoxic effect on either of the strains (Figure 5), although 1,3-DAP diminished the growth rate in both WT and Δ*Bcnrps*1 strains.

Our results indicate that deleting the *Bcnrps*1 gene did not affect the mycelial development of the fungus; however, the metabolic activity of the conidia (resazurin reduction) was reduced by 70% in the presence of SPD. Based on this finding, the rest of the phenotypic characterization of the mutant was carried out using only culture media supplemented with SPD since it was the only PA that induced a statistically significant difference between strains.

#### 3.4.2. Effect on the Production and Excretion of Siderophores

**Figure 6:**
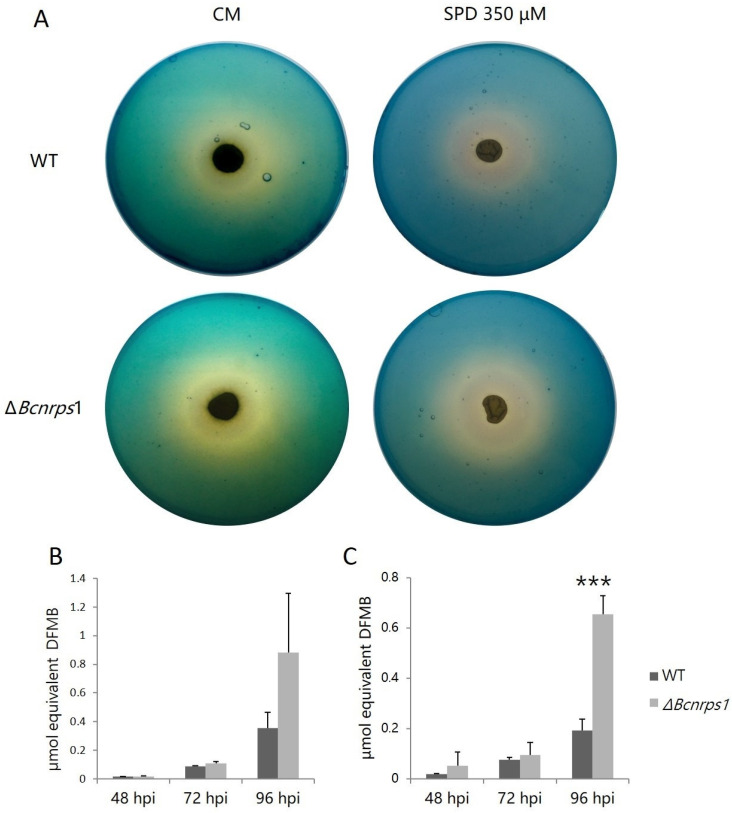
(**A**) Siderophores excretion assay on plates with CAS blue agar with *B. cinerea* B05.10 (WT) and Δ*Bcnrps*1 strains cultivated on complete medium (CM) and CM supplemented with 350 µM SPD for 96 h at 25 °C under darkness. Equivalent of siderophore standard DFMB excreted by *B. cinerea* WT (gray) and the Δ*Bcnrps*1 strain (light gray) cultivated on (**B**) CM medium and (**C**) CM medium supplemented with SPD 350 µM at 25 °C under darkness. Error bars represent standard deviation of means (*n* = 3). Asterisks indicate significant differences between WT and Δ*Bcnrps*1 strains in each condition, according to two-way ANOVA, post hoc Tukey’s HSD test for factors strain and hours post inoculation (hpi) (*p*-value < 0.001, ***).

To test the role of *Bcnrps*1 in siderophore biosynthesis, we estimated excreted siderophores using the two-layer CAS agar assay. After culturing WT and Δ*Bcnrps*1 strains, the formation of the yellow halo around the growth zone of both fungal strains was observed (Figure 6A). The formation of this yellow halo is indicative of the production of siderophores that remove Fe (III) from the Fe (III)-CAS-HDTMA complex, turning the color of the medium from blue to yellow. In addition, both strains produced and excreted siderophores, as evidenced by the positive reaction in the CAS test.

During the growth of the WT and mutant strains, siderophore excretion increased both in the absence (Figure 6B) and presence (Figure 6C) of SPD. At 48 and 72 hpi, no significant differences were found between the amount of siderophores excreted by the two strains regardless of the addition or not of PA to the culture medium. At 96 hpi, the WT strain excreted 0.4 ± 0.1 µmol DFMB equivalent growing on CM and 0.2 ± 0.05 µmol on CM with SPD versus the mutant strain, which excreted 0.9 ± 0.4 µmol and 0.7 ± 0.1 µmol equivalent DFMB, respectively. Consequently, the amount of siderophores excreted by the mutant strain was two times higher than the WT strain (Figure 6B,C) in both assays (CM *p*-value with SPD < 0.001).

This observation led us to hypothesize that the deletion of the *Bcnrps*1 gene affected the expression of the other NRPS family genes coding for siderophore synthesis. The *Bcnrps*6 gene encodes an extracellular siderophore of the coprogene family. Therefore, an expression study of the *Bcnrps*6 gene was performed to verify the increased excretion of siderophores as a consequence of the differential expression of this gene. The RT-qPCR assay demonstrated that the deletion of the *Bcnrps*1 gene affects the level of expression of the *Bcnrps*6 gene, the latter being significantly overexpressed in the mutant strain relative to the WT.

#### 3.4.3. Sensitivity Test to Oxidative Stress and Exogenous Toxic Substances

**Figure 7:**
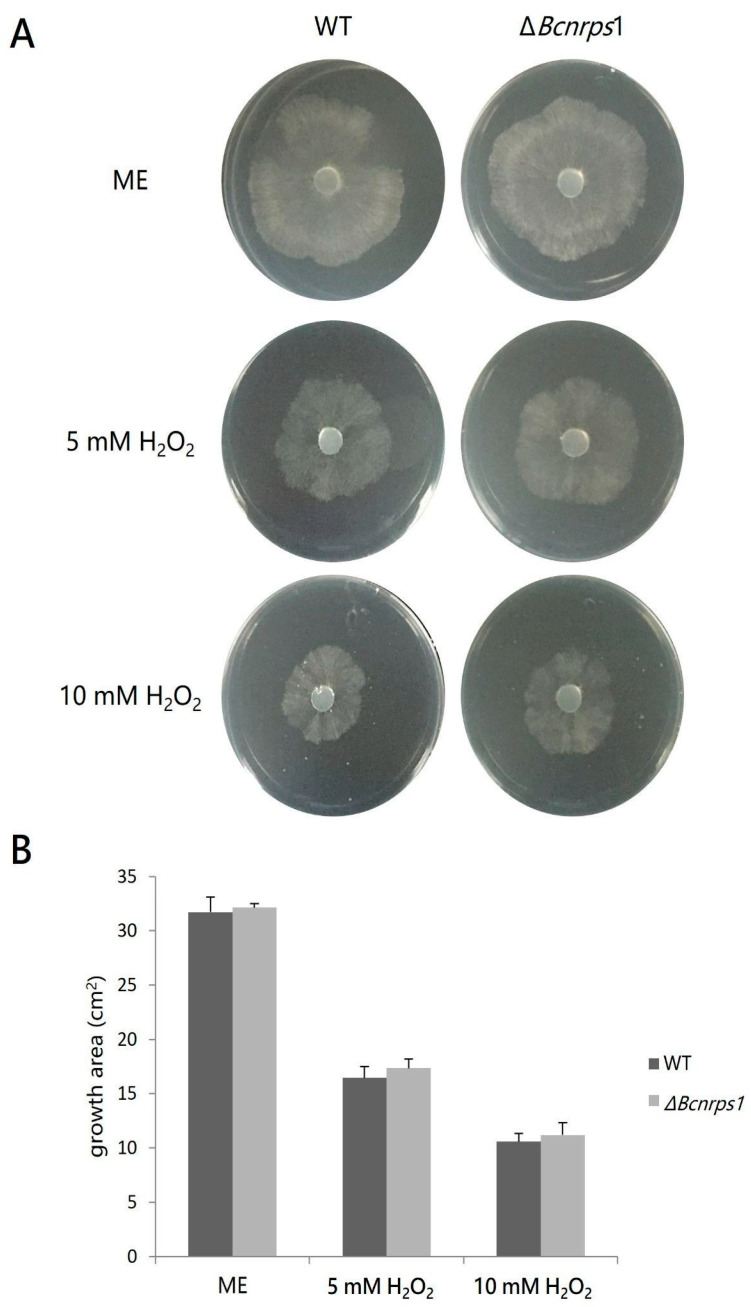
(**A**) Effect of hydrogen peroxide 4 dpi on the growth *B. cinerea* B05.10 (WT) strain and Δ*Bcnrps*1 mutant cultivated on malt extract (ME) medium without and with an increasing amount of hydrogen peroxide (H_2_O_2_) (5 or 10 mM). (**B**) Growth area of the *B. cinerea* B05.10 (WT) (gray) and Δ*Bcnrps*1 (light gray) on oxidative stress-inducing media. ME: growth area in 0.2% ME medium; 5 mM H_2_O_2_ and 10 mM H_2_O_2_: growth area in 0.2% ME amended with hydrogen peroxide 5 and 10 mM, respectively. Error bars represent standard deviations of means (*n* = 3).

A comparative study of the effect on mycelial growth in both strains in the presence of two different concentrations of hydrogen peroxide was carried out to evaluate the role of the *Bcnrps*1 gene in oxidative stress tolerance. The presence of hydrogen peroxide (5 mM and 10 mM) caused a decrease in the mycelial growth area of both the WT and mutant strains (Figure 7A). However, no statistically significant differences (*p* > 0.05) were found in the growth area of the mutant strain against WT (Figure 7B).

These results demonstrate that the *Bcnrps*1 gene is not involved in oxidative stress tolerance, which is a function previously described for this family. However, the metabolic capacity of the conidia of the mutant strain was drastically reduced in the presence of SPD (350 µM), which led us to believe that the function of this gene must be related to tolerance to exogenous toxic substances. To test this hypothesis, both strains were tested for tolerance to the fungicide pyrimethanil, a fungicide commonly used in agriculture to control *B. cinerea* and believed to inhibit the biosynthesis of methionine and other amino acids as well as the secretion of hydrolytic enzymes involved in the infection process of this phytopathogen [29].

**Figure 8:**
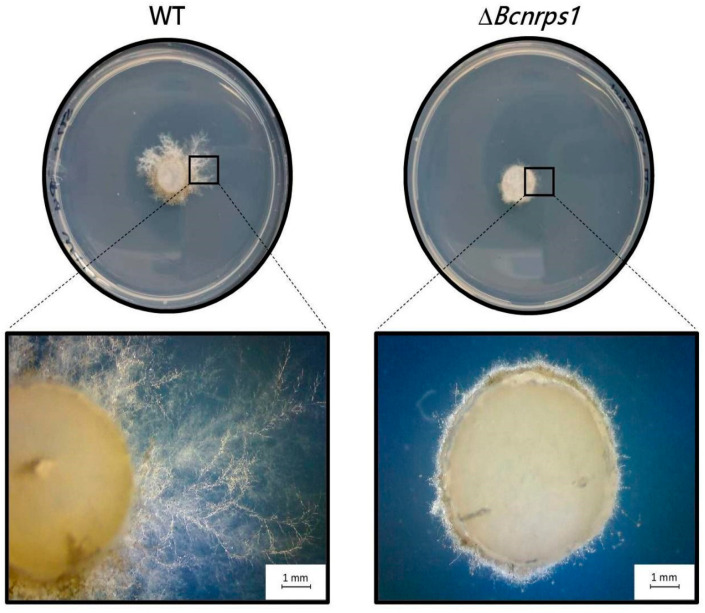
Growth of *B. cinerea* strain (WT) and Δ*Bcnrps*1 strain in SM culture medium supplemented with 1 µg/mL pyrimethanil at 3 dpi. Visual examination of the inoculation zone of the strains by observation under binocular microscope.

After 3 days of incubation in SM medium supplemented with 1 µg/mL pyrimethanil, a total reduction in the growth of the mutant strain was observed compared to the WT strain (Figure 8), showing that the function of the *Bcnrps*1 gene is to protect the *B. cinerea* fungus against some exogenous toxic substances.

#### 3.4.4. Pathogenicity Assays

**Figure 9:**
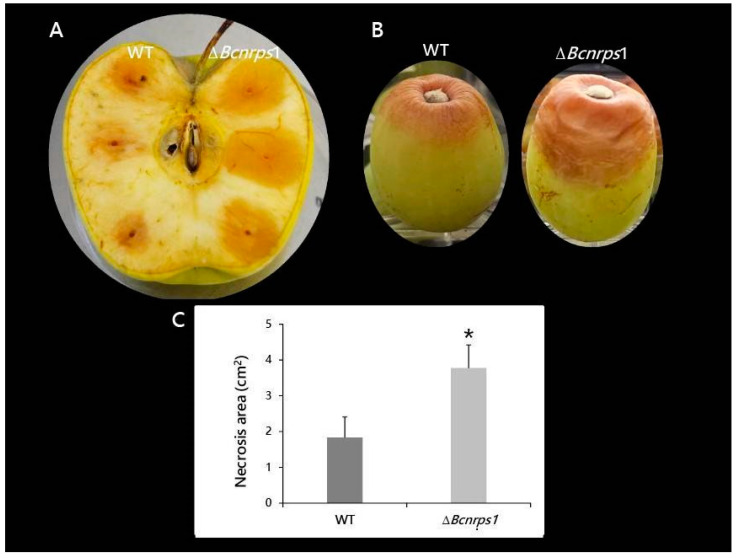
Pathogenicity test of *B. cinerea* B05.10 (WT) and Δ*Bcnrps*1 strains in wounded pieces of (**A**) apple at 4 dpi and (**B**) white grape berries after 9 dpi. (**C**) Area of necrosis produced in apple by the strains at 4 dpi. Error bars represent standard deviations of means (*n* = 3). Asterisks indicate significant differences between strains, according to the *t*-test (*p*-value < 0.05, *).

Both strains, *B. cinerea* B05.10 (WT) and Δ*Bcnrps*1 were able to infect both apples and grapes; however, differences in the size of necrotic lesions caused by the strains were observed (Figure 9). The area of necrosis was significantly larger, 4 dpi, when the mutant infected the apple (Figure 9A,C). At 6 dpi, the mutant strain had colonized the whole apple and produced conidiophores, while in the WT-infected apple, the limits of necrotic lesions were still visible on the fruit. Similar effects were recorded on grapes, 9 dpi, where 66% of berries inoculated with the mutant strain showed 50% of their fruit surface necrotic (Figure 9B). In contrast, only 11% of berries infected with the WT strain showed such an effect.

## 4. Discussion

Polyamines are ubiquitous molecules widely distributed in nature. Indeed, they are present in all cells, including prokaryotes and eukaryotes. Their function is to regulate different cellular processes such as growth, gene expression, or cell death. During the process of infection of the grapevine, *Vitis vinifera*, by *B. cinerea*, intracellular PAs levels in the plant rise in response to biotic stress [24,25,26]. PAs have previously been used as activators to synthesize NRPS metabolites such as penicillin [27]. However, the molecular response of *B. cinerea* to PAs is still unknown, as is the function of its NRPS genes. Thus, we decided to study the effect of PAs (1,3-DAP, SPD, and SPM) on the expression of the nine genes of the NRPS family. For this purpose, experiments were carried out following a One Strain Many Compounds (OSMAC) approach [48]. Fermentations of *B. cinerea* B05.10 strain in culture medium supplemented with sublethal concentrations of PAs were performed, and the influence of these substances on the expression of NPRS was analyzed. Previous studies in other filamentous fungi have shown that NRPS functions include resistance to oxidative stress [10], synthesis of bioactive molecules, and iron uptake and storage [7,8,9]. Of the nine genes that code for this type of enzyme in *B. cinerea*, the function of only four of them, *Bcnrps*2, *Bcnrps*3, *Bcnrps*6, and *Bcnrps*7, has been described.

While PAs are necessary compounds for several vital processes, they can also have a potent cytotoxic effect, and therefore their intracellular homeostasis must be tightly regulated. After testing the effect of increasing amounts of the three PAs, 1,3-DAP, SPM, and SPD, on the growth of *B. cinerea* B05.10, we determined that the maximum sublethal concentrations were 1.5 mM, 10 µM and 350 µM for 1,3-DAP, SPM, and SPD, respectively. Of note, the concentrations of SPM and SPD were much lower than those of 1,3-DAP, indicating that these two polyamines, particularly SPM, have a substantial potent toxic effect on the fungus under the conditions of our study. Our findings are consistent with previous studies testing the antibacterial capacity of PAs against *Staphylococcus aureus* and *Escherichia coli*. The most potent antibacterial PA was also SPM for both bacteria, at a concentration of just 9.88 µM, similar to the SPM concentration determined in our study (10 µM) [49]. However, other filamentous fungi, such as *Pyrenophora avenae* [50], *Penicillium chrysogenum* [27,51], and *Aspergillus terreus* [52], have a higher tolerance than *B. cinerea* to exogenous PAs. In those cases, fungi were able to tolerate concentrations of 1 mM of SPM in *P. avenae* and up to 5 mM of 1,3-DAP and SPD in cultures of *P. chrysogenum* or *A. terreus*. Consequently, it is paramount to optimize the minimum inhibitory concentration of each PA for each fungus studied.

Adding sublethal concentrations of each PA to the culture medium resulted in changes in the expression profiles of the nine *Bcnrps* genes (Figure 2B–D) during 22 days of fermentation. The most significant effect was the overexpression of the *Bcnrps*1 gene at 19, 4 and 9 dpi in medium supplemented with SPM (Figure 2B), SPD (Figure 2C), and 1,3-DAP (Figure 2D), respectively.

This stimulating effect of PAs on the expression of *nrps* genes has been observed previously in other filamentous fungi. For example, in previous reports, cultures of *P. chrysogenum* [27,51] or *A. terreus* [52] had been supplemented with PAs to activate the penicillin and lovastatin biosynthetic pathways, which are secondary metabolites belonging to the non-ribosomal peptide family. This activation of the biosynthetic pathways is due to 1,3-DAP and SPD maintaining the universal regulator LaeA in *P. chrysogenum* and *A. terreus* high. The LaeA regulator was first identified in *A. nidulans* [53,54] and forms a protein VELVET complex responsible for regulating the different biosynthetic pathways of secondary metabolism in filamentous fungi [55]. Furthermore, Schumacher et al. [56] observed that the deletion of *BcLaE*1 in *B. cinerea*, an ortholog of LaeA, decreased the level expression of the *Bcnrps*1 in *B. cinerea* B05.10. Therefore, it would be interesting to know the potential effect of SPD on the *BcLaE*1 gene expression in this fungus.

To elucidate the function of the *Bcnrps*1 gene in the life cycle of *B. cinerea*, we deleted this gene in the *B. cinerea* B05.10 strain. The behavior of the mutant strain was compared with the WT in the different cellular processes in which the involvement of NRPS has been described, such as pathogenicity, resistance to oxidative stress, siderophore production, and synthesis of bioactive molecules [7,8,9,10]. Deleting the *Bcnrps*1 gene did not result in differences in the growth rate of neither the wild-type *B. cinerea* B05.10 strain nor the mutant strain on ME medium with or without added PAs (Figure 5). However, the metabolic capacity of the conidia of the mutant strain was drastically reduced in the presence of SPD (350 µM) (Figure 4).

Regarding the resistance to oxidative stress, our data show that the *Bcnrps*1 gene does not play a role in the survival of *B. cinerea* (Figure 7). The growth rate of the mutant strain was similar at 5 and 10 mM hydrogen peroxide compared to the WT strain. This result is contrary to that observed in the pathogen *A. fumigatus*, where deletion of the *Pes*1 gene, which encodes an NRPS, resulted in a significant increase in sensitivity to oxidative stress, in addition to a reduction in virulence [57].

To understand the implication of the gene in the synthesis of bioactive molecules, fermentations were carried out with the strains following the OSMAC methodology. However, the concentration of metabolites isolated from the fermentations supplemented with SPD was too low, which prevented the isolation and characterization of the putative metabolite synthesized by NRPS1 enzymatic complex in *B cinerea* under the study conditions.

**Figure 10:**
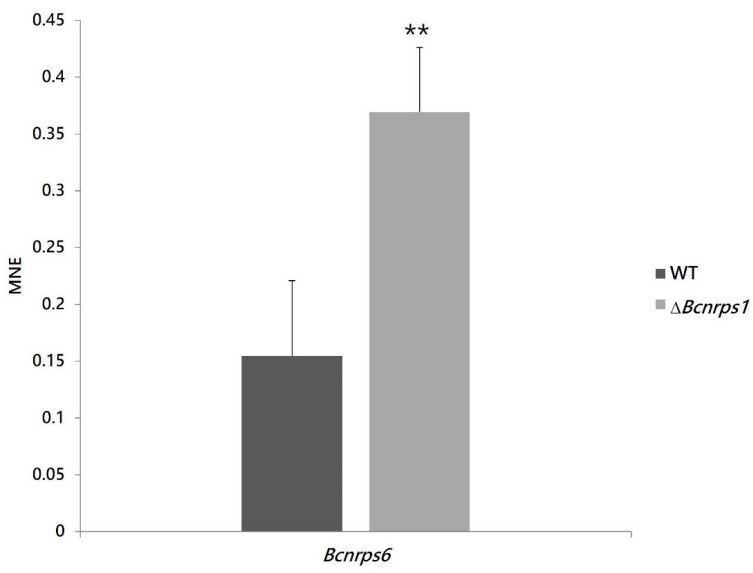
*Bcnrps*6 gene expression profile in *B. cinerea* B05.10 (WT, gray) and ∆*Bcnrps*1 (light gray) 96 hpi in CM medium. The mean normalized expression (MNE) represents an average of the two MNE estimated using actin and β-tubulin as reference genes. Error bars represent standard deviations of means (*n* = 3). Asterisks indicate significant differences between strains, according to *t*-test (*p*-value < 0.01, **).

Among the functions described for genes coding for NRPS are siderophore synthesis and fungal virulence. In the present study, we confirm that the *Bcnrps*1 gene is not involved in siderophore synthesis since deletion of this gene resulted in a significant increase in siderophore excretion at 96 hpi (Figure 6). In addition, the *Bcnrps*6 gene was overexpressed in the Δ*Bcnrps*1 strain (Figure 10, leading to increased excretion of coprogen, a siderophore synthesized by the NRPS6 gene in *B. cinerea*. In the necrotrophic fungus *Alternaria alternata* [58], deletion of the *nrps*6 gene, responsible for dimethyl coprogen biosynthesis, led to reduced iron absorption efficiency and increased sensitivity to oxidative stress [58]. The *nrps*6 gene is conserved among different plant pathogenic fungi belonging to Ascomycetes [13], including *B. cinerea* [14,15]. Considering that the mutant strain shows the same resistance to hydrogen peroxide as the WT, these results suggest that the overexpression of the *Bcnrps*6 gene in the mutant strain contributes to its survival to oxidative stress.

In terms of fungal pathogenicity, the wild and mutant strains behaved differently. The areas of necrotic lesions were higher in both fruits (apple and grape) when infected with the ∆*Bcnrps*1 strain than the WT (Figure 9). One of the first symptoms of a *B. cinerea* infection is necrotic lesions resembling the ones produced by the hypersensitive plant response. Govrin and Levin [59] found that *B. cinerea* growth in the *Arabidopsis* plant is enhanced by the elevated generation of reactive oxygen intermediates (ROIs) and subsequent increase in hypersensitive cell death in the plant. In addition, Tiedemann [60] showed that the highest levels of ROIs occur in plants infected by more aggressive strains of *B. cinerea*. Therefore, the increase in fruit necrosis after infection by the Δ*Bcnrps*1 strain is caused by the higher aggressiveness of this strain, which triggered local cell death. The above results show that the *Bcnrps*1 gene is not essential for the infection process of *B. cinerea*, but its deletion increased the infective capacity of the *B. cinerea* B05.10 strain.

The addition of SPD resulted in the overexpression of the *Bcnrps*1 gene in the WT strain and a decrease in metabolic activity up to 70% when the mutant grew in the presence of it (Figure 4). This finding could be attributed to the fact that the *Bcnrps*1 gene exerts a protective effect against exogenous toxic substances. To corroborate this notion, we sought to determine the sensitivity of the mutant strain to the fungicide pyrimethanil. We found that a sublethal concentration of the fungicide (1 µg/mL) for the WT strain inhibited the growth of the mutant strain (Figure 8). In light of these data, we conclude that the *Bcnrps*1 gene protects *B. cinerea* against certain exogenous toxicants such as pyrimethanil and spermidine.

In conclusion, our results based on the induction of *Bcnrps* gene expression and the implementation of both experimental and bioinformatics approaches show that the presence of PAs in *B. cinerea* culture triggers an increase in the expression of the *Bcnrps*1 gene, with no overexpression of the other NRPS genes. Although our experimental setup could not determine the metabolite produced by the *Bcnrps*1 gene due to its reduced scale, a protective function in the fungus against exogenous toxic substances (SPD and pyrimethanil) could be inferred for this gene. In addition, it was established that it is not essential for the infection cycle of the phytopathogen and reduces the aggressiveness of the *B. cinerea* B05.10 strain. Although we have optimized our experimental conditions for the overexpression of the *Bcnrps*1 gene, the complexity and versatility attributed to the metabolism of *B. cinerea* call for the search for other appropriate conditions that allow the functional analysis of the other genes of the NRPS family.

## Data Availability

Data is contained within the article or supplementary material.

## Supplementary Materials

The following are available online at https://www.mdpi.com/article/10.3390/jof7090721/s1, Table S1. Information from the database of *Bcnrps* genes and specific primer sequences used in the expression study of the *Bcnrps* genes in *B. cinerea*, Table S2. Specific primers sequences for the generation of *Bcnrps*1 mutants and the diagnostic of *Bcnrps*1 homokaryotic mutants, Table S3. Standard curve efficiency data for each *Bcnrps* gene and each reference gene used in the RT-qPCR assay, Table S4. Analysis of *Bcnrps*1 orthologous genes in Ensembl Fungi Database, Table S5. Proteins sequences with significant alignment in BLASTP analysis, Table S6. Conserved domains in NRPS1 proteins in Conserved Domain Database (CDD) of NCBI and Ensembl Fungi Database. Figure S1. Expression profiles of the nine *Bcnrps* genes in *B. cinerea* strain B05.10 during fermentation in 0.2% malt extract (ME) with or without 350 μM spermidine (SPD) supplement, Figure S2. Expression profiles of the nine *Bcnrps* genes in *B. cinerea* strain B05.10 during fermentation in 0.2% malt extract (ME) with or without 1.5 mM 1,3-Diaminopropane (1,3-DAP) supplement, Figure S3. Expression profiles of the nine *Bcnrps* genes in *B. cinerea* strain B05.10 during fermentation in 0.2% malt extract (ME) with or without 10 μM spermine (SPM) supplement, Figure S4. Verification of the homologous integration of the replacement fragment and the absence of *Bcnrps*1 alleles in two independent *B. cinerea* strains by means of a 1% (*w*/*v*) agarose gel electrophoresis. The amplification product to verify the homologous integration at 3′ (1595 pb) and at 5′ (883 pb). The transformants T1.2 and T1.4 displayed the expected PCR products for homologous integration of the KO construct, whereas the *Bcnrps*1 allele was only amplified in the *B. cinerea* WT strain (427 pb). M: GeneRuler™ 1kb DNA Ladder.
