# Supplementary material for: Deletion of the Bcnrps1 Gene Increases the Pathogenicity of Botrytis cinerea and Reduces Its Tolerance to the Exogenous Toxic Substances Spermidine and Pyrimethanil
Source: J Fungi (Basel). 2021 Sep 3;7(9):721. doi: 10.3390/jof7090721 (PMC8467525; doi:10.3390/jof7090721)
Supplement: Supplementary file 1 [file jof-07-00721-s001.zip › jof-1337840-supplementary.pdf]

Table S1. Information from the database of *Bcnrps* genes and specific primer sequences used in the expression study of the *Bcnrps* genes in *B. cinerea*.

	EnsemblFungi Gene ID	NCBI Gene ID	NCBI Genomic Sequence	NCBI Protein Accession	Primer	Sequence 5'-3'	Reference
Bcnrps1	Bcin12g04980	BC1G_07441-2.1<br>ID: 5434423	NW_001814530.1	EDN27676.1	*Bcnrps*1-Fwd<br>*Bcnrps*1-Rev	CACCTCAGATCAAGACAGTC<br>GCATCGTGTTTCTAGAGAGG	Designed in this study
Bcnrps2	Bcin12g00690	BC1G_03511.1<br>ID: 5438503	NW_001814559.1	EDN20121.1	*Bcnrps*2-Fwd<br>*Bcnrps*2-Rev	GCCCAAGATGAGAAGAAGAC<br>CGAACTGCATTACTAGGTGC	Designed in this study
Bcnrps3	Bcin16g03570	BC1G_10927-8.1<br>ID: 5431255<br>ID: 5431256	NW_001814497.1<br>NW_001814497.1	EDN31726.1<br>EDN31727.1	*Bcnrps*3-Fwd<br>*Bcnrps*3-Rev	CCATCATACATGTGTCCCTC<br>CAAAGTGCTTCTGTCATCGC	Designed in this study
Bcnrps4	Bcin02g02380	BC1G_02495.1<br>ID: 5439495	NW_001814564.1	EDN32118.1	*Bcnrps*4-Fwd<br>*Bcnrps*4-Rev	CGTTTGTCCGATGCAAACAC<br>CGTGGACACAAGAATGAACC	Designed in this study
Bcnrps5	Bcin04g01390	BC1G_10622.1<br>ID: 5431399	NW_001814499.1	EDN31562.1	*Bcnrps*5-Fwd<br>*Bcnrps*5-Rev	GGTCTAATGATGTCGCTCTG<br>GGTTTTCCAAGATTCGAGCC	Designed in this study
Bcnrps6	Bcin01g03730	BC1G_10566-7.1<br>ID: 5431508<br>ID: 5431509	NW_001814500.1<br>NW_001814500.1	EDN31506.1<br>EDN31507.1	*Bcnrps*6-Fwd<br>*Bcnrps*6-Rev	CACAATACACTGGAAGCGAC<br>CACTGTTGCGATCAATGGAC	Designed in this study
Bcnrps7	Bcin01g11450	BC1G_15493.1<br>ID: 5426468	NW_001814392.1	EDN22576.1	*Bcnrps*7-Fwd<br>*Bcnrps*7-Rev	CTCCATTGGAGAAGTTGCAG<br>GATTCTGTGATAGCGTTGCC	Designed in this study
Bcnrps8	Bcin11g02650-60	BC1G_04782.1<br>ID: 5437361	NW_001814552.1	EDN23513.1	*Bcnrps*8-Fwd<br>*Bcnrps*8-Rev	CAGATGTCTGATGGAACCTG<br>GAACAGAGGTTGTCCAAACG	Designed in this study
Bcnrps9	Bcin14g01300	BC1G_09040.14I<br>D: 5433383<br>ID: 5433384	NW_001814520.1<br>NW_001814520.1	EDN29094.1<br>EDN29095.1	*Bcnrps*9-Fwd<br>*Bcnrps*9-Rev	CAGAAGCTCAAAGCTCTGTG<br>CCACCAGTTTGATAGCTAGG	Designed in this study
BcactA	Bcin16g02020	BC1G_08198<br>ID: 5433899	NW_001814525.1	EDN28275.1	*Bcact*-Fwd<br>*Bcact*-Rev	TTTGAGACCTTCAACGCCCC<br>ACGTGAGTAACTCCGTCACC	[28]
BctubA	Bcin01g08040	BC1G_00122<br>ID: 5441652	NW_001814571.1	EDN17544.1	*Bctub*-Fwd<br>*Bctub*-Rev	TCCTTTCGGTCAACTCTTCCG<br>CACCCTCAGTGTAATGACCC	[28]

Table S2. Specific primers sequences for the generation of *Bcnrps*1 mutants and the diagnostic of *Bcnrps*1 homocaryotic mutants.

Primer	Sequence 5'-3'	Purpose
*Bcnrps*1-3F	ctccttcaatatcatcttctgtctccg-GCATCTACCAACTATTTCGG	Amplification of 3' flank
*Bcnrps*1-3R	gcggataacaatttcacacaggaaaca-ATATACAATACTCGCAGGGG	Amplification of 3' flank
*Bcnrps*1-5F	gtaacgccagggttttcccagtcacgacg-CTTCCCAACGAAAGATTGCG	Amplification of 5' flank
*Bcnrps*1-5R	atccacttaacgttactgaaatctccaac-ATTTCTCCAATCACACCCGG	Amplification of 5' flank
hphF-trpC-P	GTCGGAGACAGAAGATGATATTGAAGGAGC	Amplification of the resistance cassette
hphR-trpC-T	GTTGGAGATTTCAGTAACGTTAAGTGGAT	Amplification of the resistance cassette
TrpC-P2	CCTCCACTAGCTCCAGCCAAGCCC	Diagnostic PCR – homologous integration at *bcnrps1*-3'
TrpC-T	GGAATAGAGTAGATGCCGACCGG	Diagnostic PCR – homologous integration at *bcnrps1*-5'
*Bcnrps*1-Hi 5'	GTATCAACTGGCTTGTTGCC	Diagnostic PCR – homologous integration at 5'
*Bcnrps*1-Hi 3'	ATGATGGATGGATGGATGGG	Diagnostic PCR – homologous integration at 3'
*Bcnrps*1-WT-F	AAGTACTCCCTGTTCATGCC	Diagnostic PCR – *Bcnrps1* allele
*Bcnrps*1-WT-R	ACTCTGTACTTTCCCAGTGG	Diagnostic PCR – *Bcnrps1* allele

Table S3. Standard curve efficiency data for each *Bcnrps* gene and each housekeeping gene using in the RT-qPCR assay.

Primer	Amplicon size (basepair)	Efficiency (%)	Slope	Y-Int	R^2
*Bcnrps*1-Fwd<br>*Bcnrps*1-Rev	111	106.3	3.18	19.482	0.994
*Bcnrps*2-Fwd<br>*Bcnrps*2-Rev	110	99.1	3.343	16.223	0.999
*Bcnrps*3-Fwd<br>*Bcnrps*3-Rev	107	98.6	3.356	16.902	0.999
*Bcnrps*4-Fwd<br>*Bcnrps*4-Rev	115	98.5	3.359	19.117	0.996
*Bcnrps*5-Fwd<br>*Bcnrps*5-Rev	106	94.2	3.47	18.536	0.998
*Bcnrps*6-Fwd<br>*Bcnrps*6-Rev	107	105.9	3.188	19.45	0.996
*Bcnrps*7-Fwd<br>*Bcnrps*7-Rev	102	95.7	3.43	18.35	0.996
*Bcnrps*8-Fwd<br>*Bcnrps*8-Rev	111	109.8	3.108	19.564	0.996
*Bcnrps*9-Fwd<br>*Bcnrps*9-Rev	115	91.2	3.552	23.91	0.998
Bcact-Fwd<br>*Bcact*-Rev	116	93	3.501	20.654	0.996
Bctub-Fwd<br>*Bctub*-Rev	92	99.8	3.327	20.727	0.996

Table S4. Analysis of *Bcnrps*1 orthologous genes in Ensembl Fungi Database

Species	EnsemblFungi Gene ID	Target % ID	Query % ID	Genomic location
Sclerotiniasclerotiorum	SS1G_08561	84.94%	89.64%	CH476631:1,161,754-1,167,703:-1
***Sclerotinia borealis* F-4128 (GCA_000503235)**	SBOR_3744	85.78%	86.37%	KI628588:177,747-182,558:1
Aspergillusniger	An09g00520	55.79%	54.21%	AM270988:95,688-100,331:-1
***Pyrenochaeta sp.* DS3sAY3a (GCA_001644535)**	IQ07DRAFT_551373	54.73%	52.70%	KV441664:14,471-19,452:-1
***Paraphaeosphaeriasporulosa* str. AP3s5-JAC2a (GCA_001642045)**	CC84DRAFT_1239987	51.85%	51.01%	KV441550:2,197,198-2,202,067:-1

Table S5. Proteins sequences with significant alignment in BLASTP analysis.

Description	Max Score	Total Score	Query Cover	E-value	Per. ident
BcNRPS1 nonribosomal peptide synthetase [*Botrytis cinerea* T4]	3306	3306	100%	0.0	99.94
putative nonribosomal peptide synthase -like protein [*Botrytis cinerea* BcDW1]	3299	3299	100%	0.0	99.75
hypothetical protein BOTCAL_0573g00010 [*Botryotinia calthae*]	3146	3146	100%	0.0	94.98
hypothetical protein BCON_0348g00080 [*Botryotiniaconvoluta*]	3139	3139	100%	0.0	94.61
hypothetical protein BELL_0576g00060 [*Botrytis elliptica*]	3139	3139	100%	0.0	94.29
hypothetical protein BPAE_0212g00170 [*Botrytis paeoniae*]	3135	3135	100%	0.0	94.42
hypothetical protein BHYA_0064g00320 [*Botrytis hyacinthi*]	3130	3130	100%	0.0	93.98
hypothetical protein BPOR_0035g00080 [*Botrytis porri*]	3127	3127	100%	0.0	94.42
hypothetical protein BTUL_0224g00100 [*Botrytis tulipae*]	3116	3116	100%	0.0	94.11
hypothetical protein BOTNAR_0113g00090 [*Botryotinianarcissicola*]	3101	3101	100%	0.0	93.54
hypothetical protein BGAL_0396g00050 [*Botrytis galanthina*]	3084	3084	100%	0.0	93.17
hypothetical protein sscle_10g078260 [*Sclerotiniasclerotiorum* 1980 UF-70]	2967	2967	99%	0.0	89.07
hypothetical protein SS1G_08561 [*Sclerotiniasclerotiorum* 1980 UF-70]	2957	2957	99%	0.0	88.99
hypothetical protein EYC84_000428 [*Moniliniafructicola*]	2909	2909	99%	0.0	87.45
hypothetical protein EYC80_001899 [*Monilinialaxa*]	2895	2895	99%	0.0	87.14
BcNRPS1, nonribosomal peptide synthetase [*Sclerotinia borealis* F-4128]	2853	2853	99%	0.0	85.71

Table S6. Conserved domains in NRPS1 proteins in Conserved Domain Database (CDD) of NCBI and Ensembl Fungi Database.

Conserved Domain Database (CDD) of NCBI						Ensembl Fungi			
**Domains**	**Name**	**Accession**	**Description**	**Interval**	**E-value**	**Source Domain**	**Accession**	**Description**	**Interval**
**Adenilation**	**A_NRPS_GliP_like**	cd17653	Nonribosomal PeptideSynthase GliP-like	580-1048	8.83e-149	-	cd17653	-	580-1048
	**AA-adenyl-dom**	TIGR01733	Amino acid adenylation domain	602-988	3.62e-108	-	-	-	-
**Condensation**	**C_NRPS-like**	cd19537	Condensation family domain	1157-1549	0.00e+00				
	**Condensation**	pfam00668	Condensation domain	1154-1525	9.29e-34	Pfam	pfam00668	Condensation domain	1155-1535
	**Condensation**	pfam00668	Condensation domain	147-557	2.45e-29	Pfam	pfam00668	Condensation domain	145-536
	**CT_NRPS-like**	cd19542	Terminal Condensation (CT)-like	148-536	3.47e-51	Gene3D	3.30.300.30	C-terminal domain	959-1052
**AMP binding**	**AMP-binding**	pfam00501	AMP-binding enzyme;	581-964	3.95e-76	Pfam	PF00501	AMP-dependent synthetase/ligase	581-964
	-	-	-	-	-	Prositepatterns	PS00455	AMP-binding	724-735
**Phosphopantetheine binding**	**PP-binding**	pfam00550	Phosphopantetheineattachment site	32-95	1.20e-06	Pfam	pfam00550	Phosphopantetheine binding ACP domain	1063-1138
	**PKS_PP**	smart00823	Phosphopantetheineattachment site	31-95	5.93e-03	Pfam	pfam00550	Phosphopantetheine binding ACP domain	34-95
**Chloramphenicol acetyltransferase-like domain superfamily**	-	-	-	-	-	Gene3D	3.30.559.10		1158-1497
	-	-	-	-	-	Gene3D	3.30.559.10		147-492
**Non-ribosomal peptide synthetase**	**PRK12316**	PRK12316	Peptide synthase; Provisional	123-1148	7.56e-111				
	**EntF**	COG1020	Non-ribosomal peptide synthetase	358-979	3.11e-98				
	**PRK05691**	PRK05691	Peptide synthase; Validated	508-1362	2.43e-56	Panther	PTHR45527	Non-ribosomal peptide synthetase	505-1519
	**EntF**	COG1020	Non-ribosomal peptide synthetase component F	1353-1524	2.10e-13				
	**entF**	PRK10252	Enterobactin non-ribosomal peptide synthetaseEntF	31-102	1.61e-04				

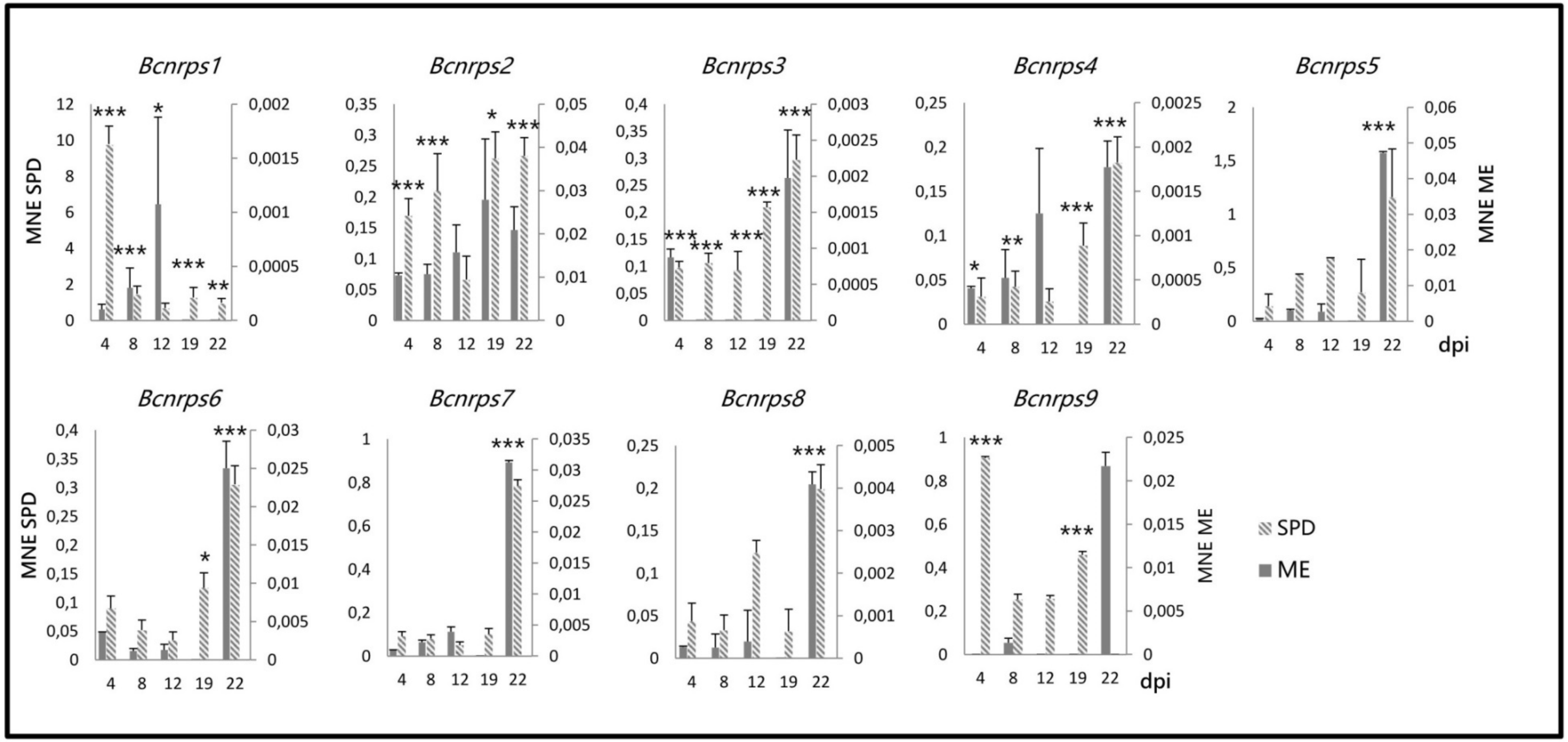

Figure S1. Expression profiles of the nine *Bcnrps* genes in *B. cinerea* strain B05.10 during fermentation in 0.2% Malt Extract (ME) with or without 350 µM Spermidine (SPD) supplement. The present Mean Normalized Expression (MNE) is an average of the two MNE estimated using *β-tubulin (BctubA)* and *actin (BcactA)* as housekeeping genes. Fermentation without 350 µM SPD supplement is represented with grey bars (ME) in the secondary axis and fermentation with 350 µM SPD (SPD) supplement is represented with striped bars in the main axis. dpi: days post inoculation. Error bars represent standard deviations of means (n = 3). Asterisks indicate significant differences between fermentations according to two-way ANOVA, post hoc Tukey's HSD test for factors media type and dpi (p-value<0.05, *; p-value< 0.01, **; p-value <0.001).

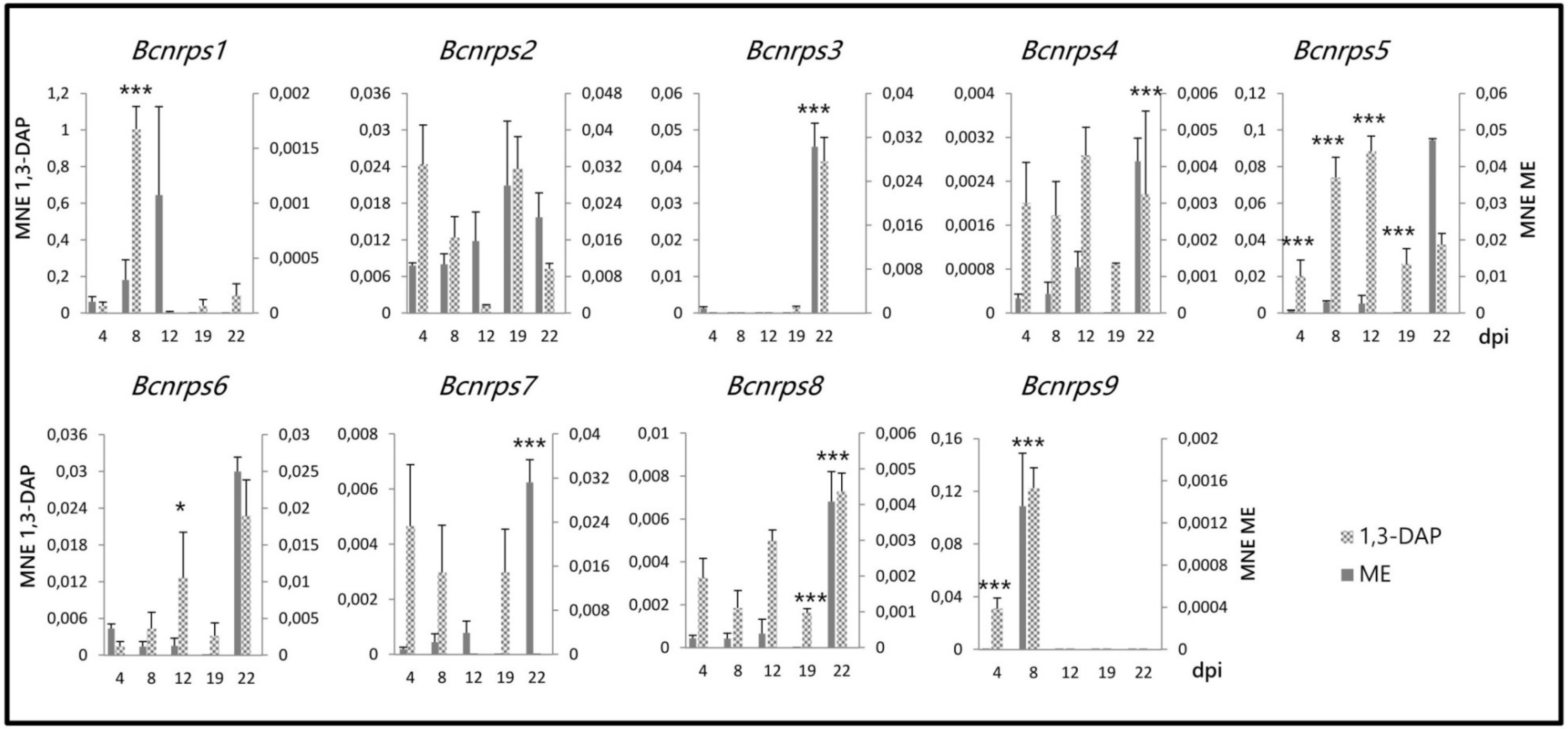

Figure S2. Expression profiles of the nine *Bcnrps* genes in *B. cinerea* strain B05.10 during fermentation in 0.2% Malt Extract (ME) with or without 1.5 mM 1,3-Diaminopropane (1,3-DAP) supplement. The present Mean Normalized Expression (MNE) is an average of the two MNE estimated using *β-tubulin (BctubA)* and *actin (BcactA)* as housekeeping genes. Fermentation without 1.5 mM 1,3-DAP supplement is represented with grey bars (ME) in the secondary axis and fermentation with 1.5 mM 1,3-DAP (1,3-DAP) supplement is represented with dotted bars in the main axis. dpi: days post inoculation. Error bars represent standard deviations of means (n = 3). Asterisks indicate significant differences between fermentations according to two-way ANOVA, post hoc Tukey's HSD test for factors media type and dpi (p-value<0.05, *; p-value< 0.01, **; p-value <0.001, ***).

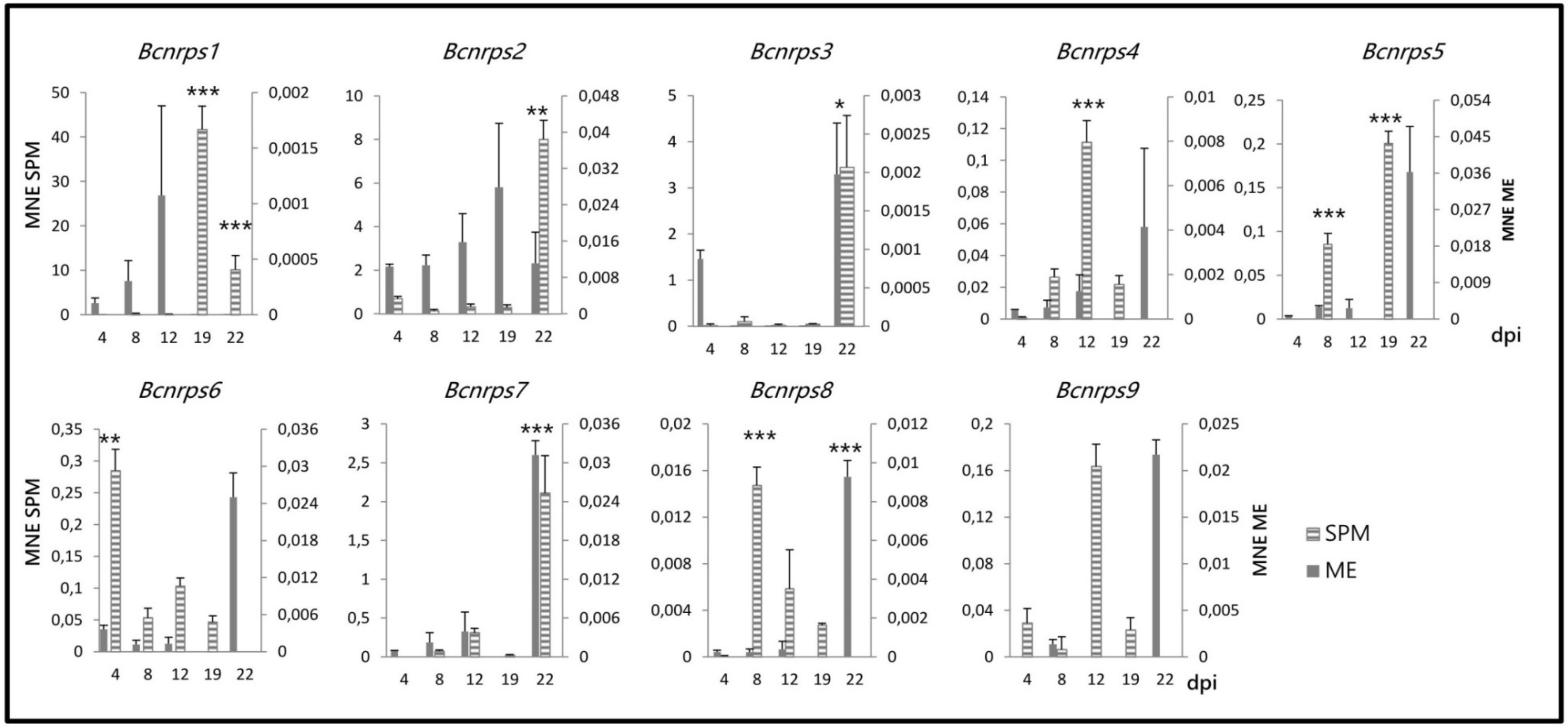

Figure S3. Expression profiles of the nine *Bcnrps* genes in *B. cinerea* strain B05.10 during fermentation in 0.2% Malt Extract (ME) with or without 10µM Spermine (SPM) supplement. The present Mean Normalized Expression (MNE) is an average of the two MNE estimated using *β-tubulin (BctubA)* and *actin (BcactA)* as housekeeping genes. Fermentation without 10µM Spermine (SPM) supplement is represented with grey bars (ME) in the secondary axis and fermentation with 10µM Spermine (SPM) supplement is represented with horizontal striped bars in the main axis. dpi: days post inoculation. Error bars represent standard deviations of means (n = 3). Asterisks indicate significant differences between fermentations according to one-way ANOVA, post hoc Tukey's HSD test for factors media type and dpi (p-value<0.05, *; p-value< 0.01, **; p-value <0.001, ***).

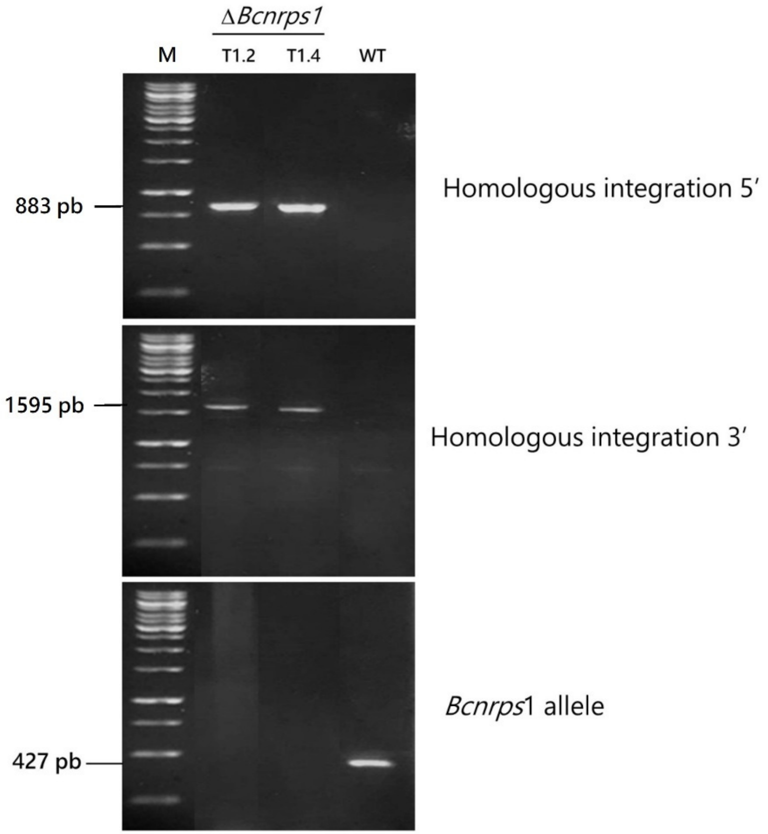

Figure S4. Verification of the homologous integration of the replacement fragment and the absence of *Bcnrps*1 alleles in two independent *B. cinerea* strains by means of a 1% (w/v) agarose gel electrophoresis. The amplification product to verify the homologous integration at 3' (1595 pb) and at 5’ (883 pb). The transformants T1.2, T1.4 displayed the expected PCR products for homologous integration of the KO construct, whereas the *Bcnrps*1 allele was only amplified in the *B. cinerea* WT strain (427 pb). M: GeneRuler™ 1kb DNA Ladder.
